# New species of the genus *Spio* (Annelida, Spionidae) from the southern and western coasts of Korea

**DOI:** 10.3897/zookeys.1070.73847

**Published:** 2021-11-15

**Authors:** Geon Hyeok Lee, Karin Meißner, Seong Myeong Yoon, Gi-Sik Min

**Affiliations:** 1 Department of Biological Sciences, Inha University, Incheon 22212, Republic of Korea Inha University Incheon Republic of Korea; 2 German Centre for Marine Biodiversity Research, Senckenberg am Meer, c/o Universität Hamburg, Martin-Luther-King-Platz 3, D-20146, Hamburg, Germany c/o Universität Hamburg Hamburg Germany; 3 Department of Biology, College of Natural Sciences, Chosun University, Gwangju 61452, Republic of Korea Chosun University Gwangju Republic of Korea

**Keywords:** Korea Strait and Yellow Sea, molecular analysis, morphology, *Spiopigmentata* sp. nov., taxonomy

## Abstract

A new spionid polychaete, *Spiopigmentata***sp. nov.**, is described from the southern and western coasts of Korea. This new species differs from its congeners by the combination of the following morphological characteristics: the presence of orange-brown pigmentation on the anterior part of the prostomium, black pigmentation on the peristomium and along the body, U-shaped nuchal organs, a comparatively long extension of metameric dorsal ciliated organs, three pairs of white dots per chaetiger, two to three posterior abranchiate chaetigers, and the presence of tridentate neuropodial hooded hooks. The partial 16S ribosomal DNA (rDNA) and nuclear 18S rDNA sequences of the new species and *Spio* sp. 2 reported by Abe and Sato-Okoshi (2021) from Japan showed high similarity, indicating that these two specimens belong to the same species. A detailed description and illustrations of the new species, together with molecular information, are provided.

## Introduction

*Spio* Fabricius, 1785 is one of the most speciose genera of Spionidae Grube, 1850. It currently comprises 37 species occurring all over the world (Read and Fauchald 2021). *Spiofilicornis* (O. F. Müller, 1776), the type species of the genus, has been repeatedly misidentified and eventually regarded as a cosmopolitan species with worldwide distribution (Okuda 1937; Imajima and Hartman 1964; Paik 1975, 1982, 1989). The confusion regarding the taxonomy of this species has been considerable, and it was evident that the identity of *S.filicornis* needed to be stabilized. Against this background, a neotype of the species from the type locality in Greenland was designated by Meißner et al. (2011), with a detailed redescription based on traditional characteristics and additional diagnostic characteristics that had been rarely or only briefly described in previous publications. They examined traditional morphological characteristics such as the shape of the anterior margin of the prostomium, length of the first branchiae, and the beginning, shape, and number of hooded hooks. They also examined additional diagnostic characteristics including the pigmentation of the body, shape of nuchal organs, extension of dorsal ciliated organs, the number and arrangement of white dots (pores of ventral epidermal glands), shape of notopodial postchaetal lamellae in the posteriormost region of the body, and the number of posterior abranchiate chaetigers (see Meißner et al. 2011). Further, molecular information regarding the three gene regions, mitochondrial cytochrome c oxidase subunit 1 (COI), 16S ribosomal DNA (16S rDNA), and the nuclear 18S ribosomal DNA (18S rDNA), also has previously been provided (Meißner et al. 2011). Thus, the re-examination of *Spio* specimens that have been identified as *S.filicornis* is now possible by additional morphological and molecular analyses, and the species can no longer be regarded as a species of worldwide distribution without scientific proof (Meißner et al. 2011).

Six *Spio* species, *S.borealis* Okuda, 1937; *S.filicornis*; *S.kurilensis* Buzhinskaya, 1990; *S.martinensis* Mesnil, 1896; S.cf.pettiboneae Foster, 1971; *S.picta* Zachs, 1933; and *S.unidentata* Chlebovitsch, 1959, have been recorded in Northeast Asia (Okuda 1937; Imajima and Hartman 1964; Wu et al. 1965; Paik 1975, 1982, 1989; Bick and Meißner 2011; Glasby et al. 2016). Okuda (1937) was the first author to report *S.filicornis* in this region, from Hokkaido Island, Japan. Imajima and Hartman (1964) later identified *Spio* specimens collected from the same area as *S.filicornis*. In Korean waters, Paik (1975, 1982, 1989) first recorded *S.filicornis* that having a prostomium with a rounded margin, a ball-shaped elevation (papillate form) on the posterior part of the prostomium, well-developed first branchiae, and bidentate hooded hooks from chaetiger 12. Characteristics later discussed by Meißner et al. (2011) were neither illustrated nor described in these publications, and hence are unknown for these specimens from Asian waters. Unfortunately, no information on deposition of Paik’s (1975, 1982, 1989) specimens had been provided.

In this study, *Spio* specimens newly collected from the southern and western coasts of Korea were examined in detail to determine the species to which they belong. An illustrated description of the new species is provided together with the partial DNA sequences of three gene regions (COI, 16S rDNA, and 18S rDNA).

## Materials and methods

### Sampling and morphological observations

**Figure 1.**
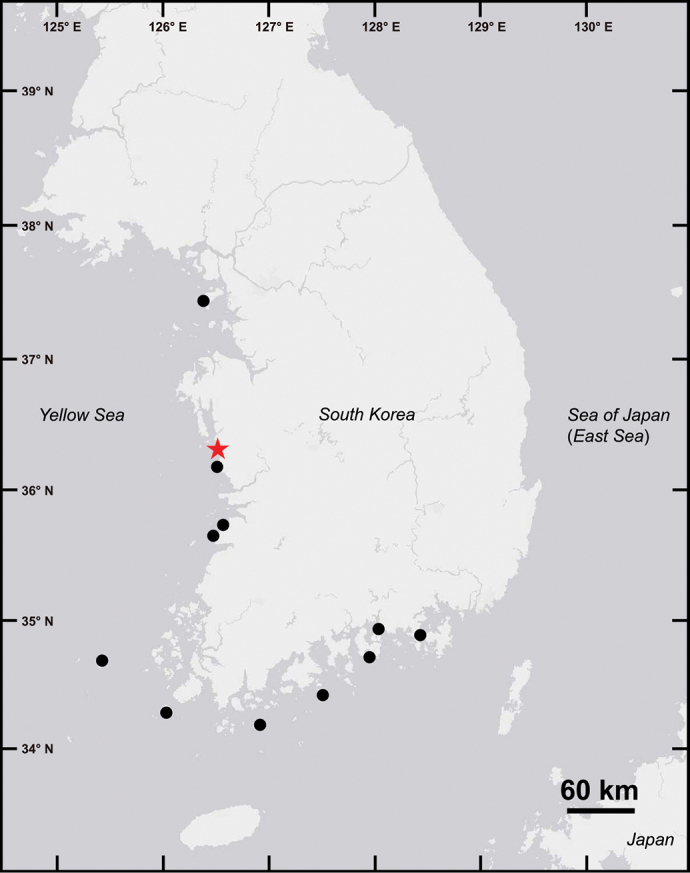
Map of the sampling locations of *Spiopigmentata* sp. nov. in this study. Type locality (red star) and collection locations of other examined specimens (circle).

Adult specimens examined in the present study were collected from the intertidal zones of the southern and western coasts of Korean waters (Fig. 1) using 500 μm-mesh sieves. The observations were performed for both live and fixed specimens. The live specimens were relaxed in 10% MgCl_2_ solution, and morphological characteristics were observed under a stereomicroscope (Leica MZ125; Germany). Photographs were taken using a digital camera (Tucsen Dhyana 400DC; Fuzhou Fujian, China) with a capture program (Tucsen Mosaic version 15; Fuzhou Fujian, China). After observation, the specimens were fixed in 4% formaldehyde for morphological analysis, washed, and subsequently transferred to 70% ethanol. For the molecular studies, the specimens were fixed with 95% ethanol. Some formalin-fixed specimens (briefly transferred to distilled water) were stained with methylene green solution to observe the pores of ventral epidermal glands, according to the method of Meißner (2005). The specimens for scanning electron microscopic examination were dehydrated using a t-BuOH freeze dryer (VFD-21S Vacuum Device; Ibaraki, Japan). The specimens were mounted on stubs and coated with gold-palladium and observations were performed using a scanning electron microscope (SU3500; Hitachi, Tokyo, Japan). Type and voucher specimens examined in this study were deposited at the National Institute of Biological Resources, South Korea (NIBR), the Senckenberg Research Institute in Frankfurt, Germany (SMF), and the Zoological Museum Hamburg, Germany (ZMH).

### Molecular analysis

Genomic DNA was extracted from the tissues of the palps of five specimens (NIBRIV0000829700–4) using a LaboPass Tissue Mini (Cosmo GENETECH, Seoul, South Korea) according to the manufacturer’s protocol. Polymerase chain reaction amplification of the partial DNA sequences of three gene regions (COI, 16S rDNA, and 18S rDNA) was performed using the following primer sets: LCO1490 and HCO709 for COI (Blank et al. 2007), 16Sar and 16Sbr for 16S rDNA (Kessing et al. 1989), and 18E and 18B for 18S rDNA (Mincks et al. 2009). Molecular analyses were performed using the partial sequences aligned using Geneious 8.1.9 (Biomatters, Auckland, New Zealand). The maximum-likelihood tree was constructed based on the concatenated partial sequences of the COI, 16S rRNA, and 18S rRNA gene regions using IQ-TREE with the GTR+F+R3 model with 1000 replicates (Kalyaanamoorthy et al. 2017; Hoang et al. 2018). The obtained DNA sequences were registered in GenBank.

## Results

### Systematics

#### Order Spionida*sensu* Rouse & Fauchald, 1997

**Family Spionidae Grube, 1850**

##### Genus *Spio* Fabricius, 1785

###### Spio pigmentata sp. nov.

Taxon classificationAnimaliaSpionidaSpionidae

A534C78A-1DF7-5111-9997-B59295B18834

http://zoobank.org/C4BA64C6-C570-4D77-A3BC-49722FC36934

Spio filicornis : Paik, 1975: 420, 1982: 808, 1989: 465, fig. 175.
Spio sp. 2: Abe and Sato-Okoshi, 2021: 63, fig. 9L–N.

####### Material examined.

**Figure 2.**
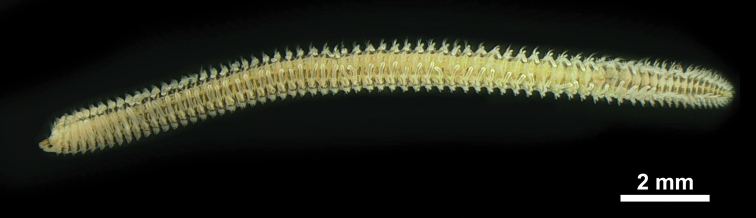
*Spiopigmentata* sp. nov., holotype, palps removed, fixed in formalin, NIBRIV0000888168.

***Type locality*.** Yellow Sea, Korea, 36°15'42.9"N, 126°32'47.9"E, intertidal sand. ***Holotype.*** Complete, without palps, formalin (NIBRIV0000888168) (Fig. 2), 21 Oct. 2020. ***Paratypes.*** Four complete (NIBRIV0000888164–7), three complete (SMF 30259), two complete (ZMH P-30424), formalin, Yellow Sea, Korea, 37°26'50.0"N 126°22'3.9"E, 13 Jan. 2021, intertidal sand.

***Non-type materials*.** Yellow Sea, Korea, intertidal sand: 27 anterior fragments (af), formalin, 34°18'43"N, 126°1'59"E, 22 Aug. 2017; 1 complete (NIBRIV0000862794), 33 af, formalin, 34°41'22.5"N, 125°25'43.8"E, 16 May. 2018; 71 af, formalin, 35°40'45.2"N, 126°31'26.5"E, 18 Mar. 2018; 14 af, 95% ethanol, 35°38'03.4"N, 126°27'57.2"E, 17 May. 2018; 5 af, 95% ethanol, 35°39'16.4"N, 126°29'26.0"E, 19 Sep. 2020; 4 af, 95% ethanol, 35°35'44.6"N, 126°29'07.9"E, 18 Sep. 2020, 5 complete, formalin, 4 af (NIBRIV0000888159–60), 95% ethanol, 35°35'44.6"N, 126°29'07.9"E, 19 Sep. 2020; 1 af, formalin, 35°40'44.2"N, 126°31'29.7"E, 21 Sep. 2020; 1 af, formalin, same locality as holotype, 21Oct. 2020; 9 complete, 1 af, formalin, same locality as paratypes, 15 Jan 2021. Korea Strait, Korea: 10 af, formalin, 34°28'05.5"N, 127°28'16"E, 26 May. 2017, intertidal sand; 3 complete, 22 af, formalin, 34°11'03.7"N, 126°54'37.5"E, 26 Jul. 2017, intertidal sand; 2 af, 95% ethanol, 34°53'19.9"N, 128°26'41.2"E, 20 Jul 2020, intertidal muddy sand; 5 af, formalin, 35°40'44.2"N, 126°31'29.7"E, 21 Sep. 2020, intertidal sand; 1 af, formalin, 2 complete (NIBRIV0000888162–3), 95% ethanol, 34°55'37.7"N, 128°02'13.3"E, 23 Jun. 2020, muddy sand between gravel and macrophytes; 8 complete, 2 af, formalin, 1 af (NIBRIV0000888161), 95% ethanol, 34°43'45.1"N, 127°57'09.7"E, intertidal sand; 1 af, formalin, 36°13'53.3"N, 126°31'47.2"E, 20 Oct. 2020, intertidal sand; 1 af, formalin, 36°09'41.2"N, 126°31'11.1"E, 20 Oct. 2020, intertidal sand.

####### Diagnosis.

Prostomium broadly rounded, slightly expanded at anterolateral margin, extending to chaetiger 1; nuchal organs with short median and long lateral ciliary bands, lateral bands extending up to transverse ciliated band (tcb) of chaetiger 3. Metameric dorsal ciliated organs double-paired, present from chaetiger 3. Branchiae from chaetiger 1 to almost end of body, length of first pair slightly shorter than that of second pair; branchiae mostly free from notopodial lamellae. White dots present from about chaetiger 3 to the end of the middle body region; three pairs of white dots per chaetiger. Neuropodial hooded hooks tridentate, present from chaetiger 11, uppermost tooth very inconspicuous. Pygidium with thin dorsolateral pair and stout but slightly longer ventral pair of anal cirri.

####### Description.

Holotype complete specimen with 67 chaetigers, about 15.7 mm in length and about 1.0 mm in width (Fig. 2). Other specimens complete with 58–73 chaetigers, 12.0–17.0 mm in length and 0.9–1.2 mm in width.

**Figure 3.**
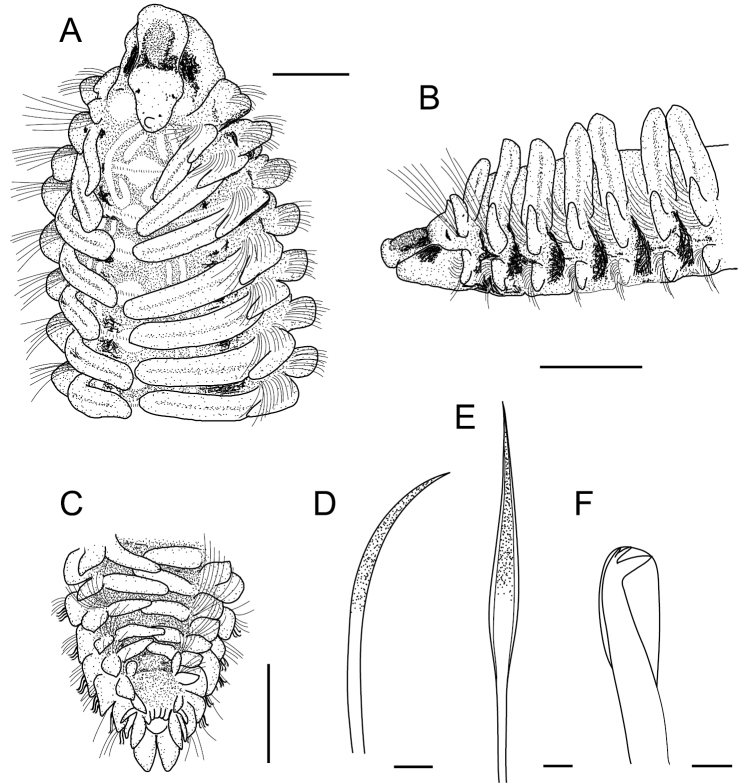
*Spiopigmentata* sp. nov. **A–C** holotype (NIBRIV0000888168) **D–F** paratype (NIBRIV0000888166) **A** anterior end, dorsal view **B** anterior end, lateral view **C** posterior end, dorsal view **D** ventral sabre chaeta from chaetiger 52 **E** anterior neurochaetae from chaetiger 22 **F** neuropodial hooded hook from chaetiger 22. Scale bars: A–C = 0.5 mm, D–F = 20.0 μm.

Prostomium entire and rounded anteriorly, slightly expanded at anterolateral margin, extending to chaetiger 1; prostomium with orange-brown pigmentation on anterior part, middle part of prostomium comparatively broad, posterior part with highly elevated papilla; two pairs of black eyes arranged in trapezoid; anterior pair larger, slightly crescent-shaped or oval, widely spaced; posterior pair smaller, rounded, closely spaced; weak transverse depression between anterior and middle part of prostomium (Figs 3A, B, 6A, 7A). Peristomium separated from prostomium by a narrow furrow (Fig. 7A). Peristomial palps reaching chaetigers 6–9 (Fig. 5A).

Nuchal organs and metameric dorsal ciliated organs distinctly observed in well-preserved and live specimens; nuchal organs U-shaped due to posterior fusion of median and lateral ciliated bands, long and recurved on chaetiger 2, and reaching to first transverse ciliated band (tcb) on chaetiger 2 (Figs 3A, 6A, 7A). Metameric dorsal ciliated organs double paired, present from between branchiae 3 and 4 (i.e., after second tcb), extending up to chaetiger 40 in holotype (38–47 in 62–73 chaetiger individuals) (Figs 3A, 5, 6A, 7A). White dots present from chaetiger 3 to chaetiger 50 in holotype (42–52 in 62–73 chaetiger individuals); three pairs of white dots per chaetiger; lateral two pairs closely spaced (Figs 5B, 6B, C, 7B). Intersegmental transverse ciliation absent.

**Figure 4.**
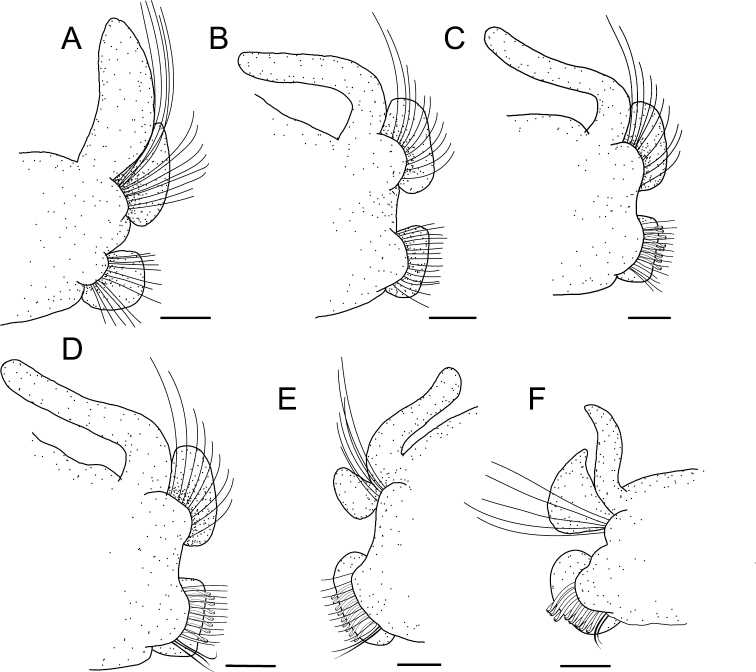
*Spiopigmentata* sp. nov., paratype (NIBRIV0000888166) **A–G** parapodium from chaetiger 1, 10, 21, 22^nd^ last, 11^th^ last, and 7^th^ last, all anterior view. Scale bars: 0.2 mm.

Branchiae present from chaetiger 1 to almost end of body, absent only on last 2 or 3 (rarely 4) chaetigers (Fig. 2); length of first pair of branchiae two-thirds to four-fifths the length of second pair (Fig. 3A, B); comparatively longest and widest branchiae on chaetigers 2–12, becoming thinner and shorter posteriorly; about last 10 branchiae distinctly shorter and thinner; branchiae with cilia on inner and furrow on outer side; branchiae mostly separated from postchaetal notopodial lamellae (Fig. 4). Notopodia on chaetiger 1 slightly shifted dorsally; notopodial postchaetal lamellae almost lanceolate (Fig. 4A); from chaetiger 2 lamellae broadly rounded, slightly tapered superiorly (Fig. 4B–D), becoming smaller in middle to posterior chaetigers (Fig. 4F), and larger, subtriangular in about last 17 chaetigers (Fig. 4G). Neuropodial postchaetal lamellae rounded in about first four chaetigers, becoming broader and larger in along anterior and middle chaetigers, largest in posteriormost chaetigers (Fig. 4).

Notopodial chaetae all capillaries; notochaetae in anterior and middle chaetigers arranged in two rows; notochaetae of anterior row with stout sheath, heavily granulated, slightly shorter than chaetae of posterior row, granulation disappearing in middle chaetigers; notochaetae of posterior row thinner, with narrow sheath, non-granulated; additional fascicle of 6–9 very long, thin capillaries without granulations present at superior position, longest in first three chaetigers; notochaetae in posterior chaetigers thin and long, arranged in irregular rows. Neuropodial chaetae with granulated or non-granulated capillaries, hooded hooks, and inferior fascicle of capillaries; capillaries of anterior neuropodia arranged in two rows; neurochaetae of anterior row with distinct sheaths, stout, heavily granulated (Fig. 3E); neurochaetae of posterior row non-granulated, less stout, replaced by 7–9 hooded hooks from chaetiger 11 (rarely 12); neuropodial hooded hooks tridentate, main fang well developed, uppermost tooth inconspicuous (Fig. 3F); inferior fascicle of 2–5 long, thin, non-granulated capillaries from chaetiger 1, replaced by 2–4 (usually 3) stout granulated, ventral sabre chaetae in inferiormost position from about chaetigers 16–19 (rarely 13–15) (Fig. 3D).

Pygidium with two pairs of anal cirri; dorsolateral pair shorter and thinner, comparatively widely spaced, and ventral pair longer, very stout, conical with rounded tip and closely spaced (Fig. 3C).

####### Pigmentation.

**Figure 5.**
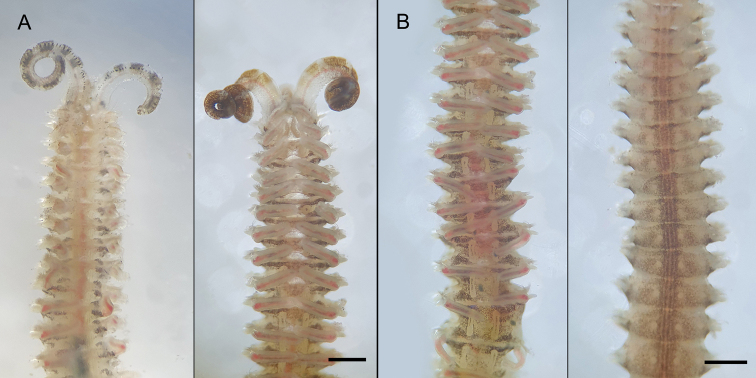
*Spiopigmentata* sp. nov., live specimens in seawater **A** specimens with palps, black-ringed (left) and dark brown (right), dorsal view **B** middle body, double-paired dorsal ciliated organs (left) and ventral white dots (right) views. Scale bars: 0.5 mm.

**Figure 6.**
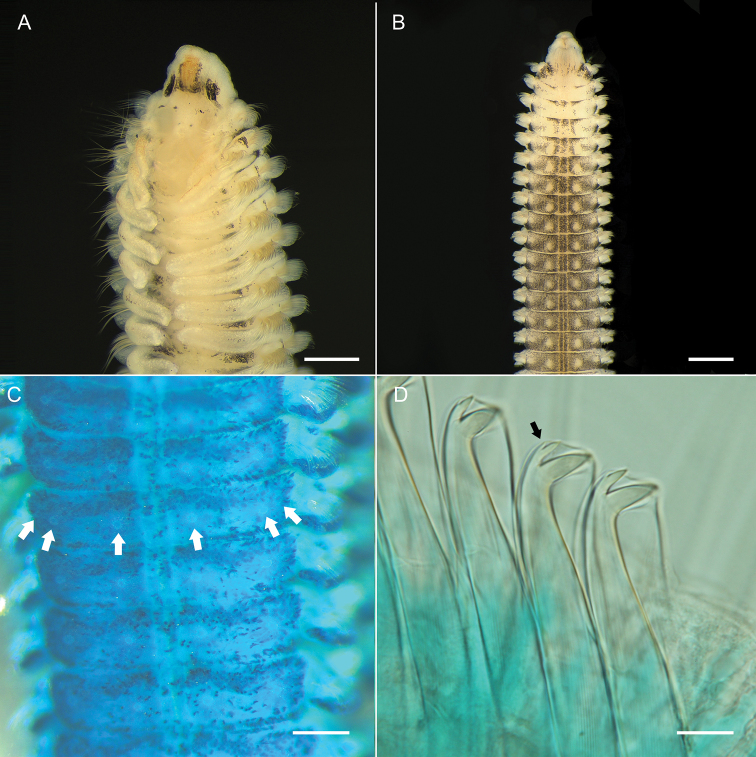
*Spiopigmentata* sp. nov. **A, B** holotype (NIBRIV0000888168), fixed in formalin **C, D** paratype (NIBRIV0000888167), fixed in formalin **A** anterior end, dorsal view **B** anterior end, ventral view **C** methyl green staining pattern of anterior end, ventral view, white dots (arrows) **D** neuropodial hooded hooks from chaetiger 15, inconspicuous uppermost tooth (arrow). Scale bars: 0.5 mm **(A–C)**; 20.0 μm **D**.

Highly variable but conspicuous in live or well-preserved specimens (some specimens without pigmentation). Palps in live specimens with variable pigmentation, about 6–15 light to dark brown spots or black ringed appearance (Fig. 5A, B); pigmentation fades in formalin- and ethanol-fixed specimens, but light brown pigmentation along the food groove remains. Well-preserved specimens with orange-brown and black pigmentation as follows: medial part of prostomium with orange-brown pigmentation, often faded in formalin- and ethanol-fixed specimens; prostomium with black pigmentation on the anterior to transverse depression margin of the prostomium, dorsal side of the peristomium next to the prostomium; black pigmented patches in front of, and in particular, behind tcb dorsolaterally in about the first six chaetigers in holotype (Fig. 6A), and some specimens with distinct patches (Fig. 5B). If black pigmented patches are distinct on the ventral side, white dots (pores of ventral glands) are clearly visible (Figs 5D, 6B).

####### Methyl green staining pattern (MGSP).

The anterior part of the prostomium and peristomium, margins of branchiae and postchaetal lamellae, and anal cirri were intensively stained. Transfer of stained specimens to distilled water for approximately 10 min, resulted in white dots being visible against the bluish background on the ventral side (Fig. 6C). Three pairs of dots were visible on chaetiger 3 to end of middle body region; lateral two pairs closely spaced, easily confused as one pair.

####### Biology.

In the present study, the specimens were found mostly in intertidal zones of fine sand, rarely muddy sand, and sometimes a mixture of gravel and macrophytes (*Zosteramarina*). According to Abe and Sato-Okoshi (2021), planktonic larvae of *Spio* sp. 2 with two rows of black melanophore spots on each side of the dorsum from chaetiger 1 onward, linked by band-shaped medial black pigmentation from chaetiger 4 or 5 are found in Sasuhama and Onagawa Bay between April and August (see cited publication for further details). Adult specimens were collected from muddy sand sediments of shallow waters in Sasuhama, Japan (Abe and Sato-Okoshi 2021).

####### Etymology.

The specific name, pigmentata, originates from the Latin word pigmentum, meaning “pigment” This name refers to the new species having conspicuous black pigmentation on the body.

####### Distribution.

Along the southern and western coasts of Korea; Sasuhama and Onagawa Bay, north-eastern Japan.

**Figure 7.**
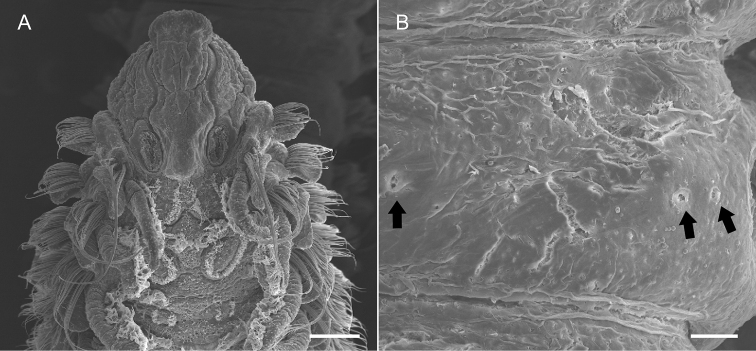
*Spiopigmentata* sp. nov., paratype (NIBRIV0000888165). **A** anterior end, dorsal view, palps removed **B** right half of middle chaetiger, ventral surface, gland openings (arrows). wScale bars: 0.2 mm **A**; 40.0 μm **B**.

### Genetics

**Figure 8.**
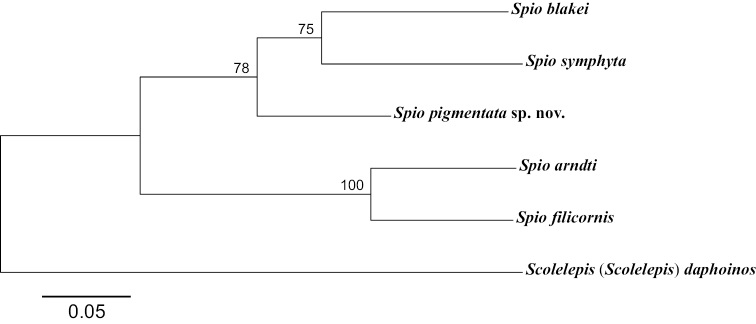
Maximum likelihood (ML) tree for 1,381 bp inferred from combined partial mitochondrial COI, 16S rDNA, and nuclear 18S rDNA from six spionid polychaetes. Numbers above the branch indicate ML bootstrap values from 1000 replication. The sequence of Scolelepis (Scolelepis) daphoinos was used for outgroup rooting.

Three DNA gene regions (COI, 16S rDNA, and 18S rDNA) from five specimens (NIBRIV0000888159–63) of the new species were determined. The lengths of each gene sequence were up to 683 bp for COI, 479 bp for 16S rDNA, and 1761 bp for 18S rDNA. The newly determined sequences have been registered in GenBank under the accession numbers MZ661756–60 (COI), MZ663825–29 (16S rDNA), and MZ663820–22 (18S rDNA). The intra-specific genetic distances between the five specimens were 0.2–1.1% for COI (666 bp) and 0.0–0.3% for the 16S rDNA (390 bp); no variation was detected with respect to the 18S rDNA (1645 bp). Pairwise genetic distances were calculated between new species and other currently available *Spio* species–*S.arndti* Meißner, Bick & Bastrop, 2011; *S.blakei*; *S.filicornis*; *S.symphyta* Meißner, Bick & Bastrop, 2011; and *Spio* sp. 2–mined from GenBank for comparison (Meißner and Götting 2015; Meißner et al. 2011; Abe and Sato-Okoshi 2021). The interspecific genetic distances between the new species and other *Spio* species were 17.2–22.6% for COI (536 bp), 5.4–17.9% for the 16S rDNA (487 bp), and 0.1–3.5% (1756 bp) for the 18S rDNA. The 16S (LC595766) and 18S rDNA (LC545924) sequences of *Spio* sp. 2 reported by Abe and Sato-Okoshi, 2021 from Japan showed 99.8% (16S: 461/462 bp) and 100.0% (18S: 1760/1760 bp) similarity with *S.pigmentata* sp. nov., indicating that these two specimens are the same species. A phylogenetic tree was constructed based on the concatenated partial gene sequences of COI (525 bp), the 16S rDNA (454 bp), and the 18S rDNA (394 bp), using maximum likelihood analyses (Fig. 8). The sequence of Scolelepis (Scolelepis) daphoinos Zhou, Ji & Li, 2009 was used as an outgroup taxon (Lee and Min 2021). The GenBank accession numbers are listed in Table 1. Phylogenetic analysis showed that two monophyletic clades were formed in *Spio*. The new species was present in a clade with *S.blakei* and *S.symphyta.*

**Table 1.** GenBank accession numbers of sequences used for phylogenetic analysis.

Species	Type locality	GenBank accession number			Data source
		COI	16S	18S	
*Spiopigmentata* sp. nov.	Korea	MZ661760	MZ663829	MZ663822	Present study
*Spioarndti*	Baltic Sea	FR823429	FR823439	FR823434	Meißner et al. 2011
*Spiosymphyta*	North Sea	FR823427	FR823437	FR823432	“
*Spiofilicornis*	West Greenland	FR823425	FR823435	FR823430	“
*Spioblakei*	Australia	KP636501	KP636502	KP636507	Meißner and Götting 2015
Scolelepis (Scolelepis) daphoinos	China	MW509617	MW494645	MW494652	Lee and Min 2021

## Discussion

Morphological examination of *Spio* specimens from the southern and western coasts of Korea, combined with the molecular analysis of three gene regions from newly collected materials, revealed the presence of a previously undescribed species of *Spio*, *S.pigmentata* sp. nov. The new species agrees well with Paik’s (1975, 1982, 1989) description of *S.filicornis* with respect to most diagnostic features and only differs in the dentation of hooded hooks (see above). The morphological examination in this study showed that the uppermost tooth in the tridentate hooks is very inconspicuous (e.g., new species in the present study and *S.symphyta* in Meißner et al. 2011). This can easily lead to erroneous conclusions. We suggest that the undescribed species newly collected during this study and the species previously known as *S.filicornis* by Paik’s (1975, 1982, 1989) from Korean waters are the same.

The new species is morphologically very similar to *S.blakei* Maciolek, 1990 from Australia in having the following characteristics: the length of first branchiae, the shape of nuchal organs, extension of dorsal ciliated organs, shape of hooded hooks, the number of abranchiate chaetigers, and the shape of the anal cirri (Meißner and Gotting 2015). However, the new species can be distinguished from *S.blakei* by the presence of three pairs of white dots per chaetiger instead of two pairs and having 7–9 tridentate hooded hooks instead of 4–5 (Meißner and Götting 2015). In the Far East of the temperate region, the new species and *S.picta* from the Kuril Islands share tridentate hooded hooks. The new species, however, differs from *S.picta* by the presence of orange-brown and black pigmentation instead of only light to dark brown, the shape of its nuchal organs (U-shaped vs straight), the number of hooded hooks (7–9 vs 8–13), and the fusion of notopodial postchaetal lamellae (mostly separated vs completely fused in the anterior and middle regions) (Bick and Meißner 2011).

According to the results of the molecular studies, the species was already recorded in Japan and published as unidentified *Spio* sp. 2 by Abe and Sato-Okoshi (2021). The known distribution of *S.pigmentata* sp. nov. ranges from the Korea Strait and the Yellow Sea of Korea to northeastern Japan.

The phylogenetic tree resulting from the analysis of molecular data revealed that *S.pigmentata* sp. nov. formed a clade with *S.blakei* and *S.symphyta* (Fig. 8). The morphological characteristics also imply a close relationship of these species. These three *Spio* species share U-shaped nuchal organs, and the two species that form a second clade (*S.arndti* and *S.filicornis*) share almost straight nuchal organs. Despite the high species diversity of the genus, the available DNA data are very poor. Further studies based on detailed morphological and molecular information of *Spio* species are needed to reveal additional information on their genetic relationships.

The identity of *Spio* species described in Northeast Asia should be verified based on both morphological and genetic studies (see Meißner et al. 2011). For example, the description of the specimens from China identified as *S.martinensis* showed morphological differences in the shape of the apical tooth of neuropodial hooded hooks and the number of posterior abranchiate chaetigers with the specimen from the type locality in France (Wu et al. 1965; Lavesque et al. 2015), and hence might be doubtful. Unfortunately, the DNA information of specimens from China and France is still unknown. Further studies are needed to resolve these and related problems.

## Supplementary Material

XML Treatment for Spio pigmentata
